
JOURNAL INFORMATION
==============================
NLM Title Abbreviation: Sci China Life Sci
Journal ID: Sci China Life Sci
NLM Journal ID: 101529880

Science China. Life Sciences

ISSN: 1674-7305
EISSN: 1869-1889

ARTICLE INFORMATION
==============================
PMCID: PMC7088778
PMID: 23881672
DOI: 10.1007/s11427-013-4529-6
Article ID: 4529
Article version: 1
Subjects: Letter to the Editor

Adenovirus sneak attacks during the H7N9 flu season

Xu Jun 14529
Li Chen 14529
Walline Joseph 24529
Yu XueZhong yxzpumch@126.com
14529
14529 grid.413106.1 0000000098896335 Chinese Academy of Medical Sciences, Peking Union Medical Hospital, Beijing, 100730 China
24529 grid.412359.8 0000000404573148 Division of Emergency Medicine, Department of Surgery, Saint Louis University Hospital, Saint Louis, Missouri 63110 USA
Electronic publication date: 2013 Jul 24
Print publication date: 2013
Volume: 56
Issue: 9
First page: 863
Last page: 864
Received 2013 Jun 12; Accepted 2013 Jul 2
Copyright: © The Author(s) 2013
License: Open AccessThis article is distributed under the terms of the Creative Commons Attribution 2.0 International License (https://creativecommons.org/licenses/by/2.0), which permits unrestricted use, distribution, and reproduction in any medium, provided the original work is properly cited.
License URL: https://creativecommons.org/licenses/by/2.0/

Keywords: H7N9 Influenza Virus, Adenovirus Infection, Adenovirus Type, Close Personal Contact, Adenovirus Vaccine
issue-copyright-statement: © Science in China Press and Springer-Verlag GmbH 2013

==============================

XU Jun[1†], LI Chen[1†], WALLINE Joseph[2] & YU XueZhong[1*]

The H7N9 influenza virus made headlines recently, with outbreaks in Chinese urban centers. These infections continue a recent trend of novel virus outbreaks, such as those involving SARS coronavirus and the H1N1 influenza virus. These outbreaks have been likened to tropical storms, and like prior 'viral storms' the latest H7N9 influenza virus has aroused public panic despite the fact that H7N9 infection rates are sporadic. At the same time, other more common ones (such as adenovirus) can lead to much more likely pathology. Recently we admitted two patients with severe adenovirus pneumonias in ARDS; both of them ICU-level care, mechanical ventilation and special positioning to maintain their oxygenation.

Adenoviruses are DNA viruses with no envelope, a double-stranded DNA genome, and a 20-sided icosahedral capsid composed of 252 capsomeres. They are widely prevalent in humans, with over 100 types identified, of which more than 60 can infect humans. Adenoviruses have caused epidemics in China since the 1950s. Most recently, adenovirus type 55 caused an outbreak in China's Shanxi Province in 2006. American scientists found in 2009 that adenoviruses could be transmitted from monkeys to humans and lead to infection. In February 2012, a viral outbreak in China's Hebei Province was confirmed as adenovirus type 55.

Unlike H7N9, adenoviruses can be spread by droplets, close personal contact or by fecal-oral routes. The general population is susceptible, in particular infants and young children, as well as the immunocompromised. Adenoviruses can infect multiple organ systems, including the respiratory, gastrointestinal, urinary, and ocular system. Its incubation period is typically 4–5 d, and clinical manifestations differ between virus types.

Adenoviral respiratory infections are common, generally mild, and self-limiting with a natural course of about 7–10 d. However, adenovirus types 7 and 55 can develop into severe pneumonias with lung consolidation (Figure 1A). Adenovirus ocular infections are often a complication of adenovirus respiratory tract infections (Figure 1B).

At present, there is no specific treatment for adenovirus infections. Symptoms of infection include fever, dry throat, cough, difficulty breathing or gastrointestinal discomfort. Therapy mainly includes non-specific antivirals, early low-dose glucocorticoids, prophylactic use of antibiotics, and organ support therapy. The lack of targeted antiviral therapy makes the prevention of adenovirus infection extremely important. The strict isolation of confirmed and suspected cases, as well as maintaining rigorous personal

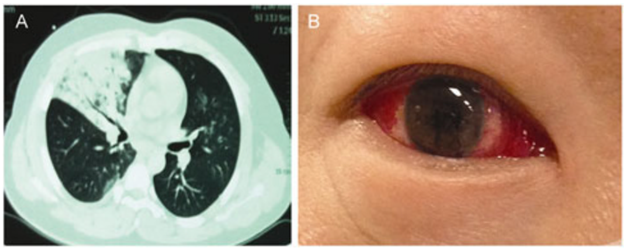

**Figure 1** CT scan of adenovirus type 7 pneumonia with lung consolidation (A) and conjunctivitis (right eye) in a doctor accidentally infected by droplets from an adenovirus-infected patient (B).

†Contributed equally to this work
*Corresponding author (email: yxzpumch@126.com)

and environmental hygiene will help to enhance resistance. Although an adenovirus vaccine containing formaldehyde-inactivated virus has been developed, it has not yet been widely used because of its apparent carcinogenicity in animal test subjects.

The infectivity and virulence of adenoviruses are high, but can be controlled. Hospitalization is often required for those with severe adenovirus infections. All medical institutions should take precautions against this virus, and maintain a high degree of vigilance for infections, especially when other viruses such as H7N9 are commanding so much attention.

Contributed equally to this work

This article is published with open access at Springerlink.com

